# Rolling out Xpert MTB/RIF^®^ for tuberculosis detection in HIV-positive populations: An opportunity for systems strengthening

**DOI:** 10.4102/ajlm.v6i2.460

**Published:** 2017-03-31

**Authors:** Ishani Pathmanathan, Anand Date, William L. Coggin, John Nkengasong, Amy S. Piatek, Heather Alexander

**Affiliations:** 1 Division of Global HIV and TB, US Centers for Disease Control & Prevention, Atlanta, Georgia, United States; 2 Epidemic Intelligence Service, US Centers for Disease Control & Prevention, Atlanta, Georgia, United States; 3 Global Health Bureau, United States Agency for International Development, Washington DC, United States

## Abstract

**Background:**

To eliminate preventable deaths, disease and suffering due to tuberculosis, improved diagnostic capacity is critical. The Cepheid Xpert MTB/RIF^®^ assay is recommended by the World Health Organization as the initial diagnostic test for people with suspected HIV-associated tuberculosis. However, despite high expectations, its scale-up in real-world settings has faced challenges, often due to the systems that support it.

**Opportunities for System Strengthening:**

In this commentary, we discuss needs and opportunities for systems strengthening to support widespread scale-up of Xpert MTB/RIF as they relate to each step within the tuberculosis diagnostic cascade, from finding presumptive patients, to collecting, transporting and testing sputum specimens, to reporting and receiving results, to initiating and monitoring treatment and, ultimately, to ensuring successful and timely treatment and cure. Investments in evidence-based interventions at each step along the cascade and within the system as a whole will augment not only the utility of Xpert MTB/RIF, but also the successful implementation of future diagnostic tests.

**Conclusion:**

Xpert MTB/RIF will only improve patient outcomes if optimally implemented within the context of strong tuberculosis programmes and systems. Roll-out of this technology to people living with HIV and others in resource-limited settings offers the opportunity to leverage current tuberculosis and HIV laboratory, diagnostic and programmatic investments, while also addressing challenges and strengthening coordination between laboratory systems, laboratory-programme interfaces, and tuberculosis-HIV programme interfaces. If successful, the benefits of this tool could extend beyond progress toward global End TB Strategy goals, to improve system-wide capacity for global disease detection and control.

## Introduction

The 2015 United Nations Sustainable Development Goals include an ambitious plan to end the tuberculosis and HIV epidemics by 2030.^1^ Yet presently, fewer than two thirds of estimated people with tuberculosis are notified, and tuberculosis remains the greatest cause of morbidity and mortality among people living with HIV.^2^ To achieve a world free of tuberculosis deaths, disease and suffering by 2035, improved tuberculosis diagnostic capacity is critical.^3^ Sputum smear microscopy, although widely used, has unacceptably poor sensitivity for detecting *Mycobacterium tuberculosis* in people living with HIV.^4,5^ Bacterial culture – the gold standard for tuberculosis diagnosis – is more sensitive, but is costly, technically challenging, and reliant on a sophisticated centralised laboratory infrastructure. Moreover, it takes weeks to obtain results, which increases delays in treatment initiation and the period of potential disease transmission or loss to follow-up.^6,7^ Efforts have therefore increasingly focused on developing rapid, near point-of-care (POC) tuberculosis diagnostic tools that can be easily utilised in resource-limited settings.^7,8,9^

In 2010, the World Health Organization (WHO) acknowledged the Cepheid Xpert MTB/RIF^®^ assay (Sunnyvale, California, United States) as a major milestone in bringing rapid, simple, bacteriologically-confirmed diagnosis of tuberculosis disease and rifampicin resistance potentially closer to the patient POC, by strongly recommending it as the initial diagnostic test for people with suspected multi-drug-resistant (MDR) or HIV-associated tuberculosis.^10,11,12,13^ A 2014 Cochrane review later determined it to be cost-effective for this indication, and verified that it significantly increases tuberculosis case detection compared with smear microscopy in people living with HIV. Pooled sensitivity was 79% and pooled specificity was 98%, and sensitivity was higher among smear-positive (97%) than among smear-negative but culture-positive people living with HIV (61%).^14^ In 2010, the United States (US) President’s Emergency Plan for AIDS Relief, the US Agency for International Development and the US Centers for Disease Control and Prevention issued a joint commitment to support the rapid and appropriate scale-up of this technology. In 2012, an agreement with Cepheid was negotiated by the US President’s Emergency Plan for AIDS Relief, the US Agency for International Development, UNITAID and the Bill and Melinda Gates Foundation to reduce the test price by 40% in 145 eligible countries. By December 2014, the public sector in these countries had procured 3763 Xpert^®^ instruments (17 883 modules) and more than 10 million MTB/RIF cartridges.^4,9,15,16,17^

Despite initially high expectations, however, rapid scale-up of Xpert MTB/RIF has uncovered limitations, many due not to the test itself, but to the systems that support it. Although initial modelling predicted that accurate same-day diagnosis by Xpert MTB/RIF could reduce tuberculosis mortality by 20%–35% by facilitating earlier treatment initiation,^18,19^ subsequent studies have failed to show a mortality benefit.^9,20,21,22,23,24^ In two multi-center, randomised-controlled trials from sub-Saharan Africa, while Xpert MTB/RIF significantly increased the proportion of tuberculosis patients starting treatment who had laboratory-confirmed diagnoses, the absolute number of patients initiating treatment remained unchanged. This was likely due to empiric treatment by clinicians with insufficient trust in the negative predictive value of available tuberculosis diagnostic algorithms^20,23,24,25,26,27,28,29^ (although notably, when Xpert MTB/RIF was located at the POC instead of a centralised laboratory, the proportion of bacteriologically-confirmed disease was higher, empiric treatment less frequent, and time to treatment shorter).^29,30^ While some studies have found that Xpert MTB/RIF availability reduces time to tuberculosis treatment initiation,^21,23,31,32,33^ others highlight persistent delays due to backlogs in machine module availability and inefficiencies in result processing and transfer.^34,35,36^ In one notable success story, decentralised Xpert MTB/RIF in a multi-country study reduced median time from sputum collection to tuberculosis treatment from 56 to five days; however, this was attributed to efficient specimen transport and result reporting systems.^37^ Finally, although predicted to be cost-effective,^13,19,38,39,40,41,42^ routinely using Xpert MTB/RIF requires ongoing investments in trained staff, supplies and infrastructure.^41,42^ Results from early programmatic implementation in nine TB REACH (http://www.stoptb.org/global/awards/tbreach/) countries revealed increased tuberculosis case detection, but also multiple challenges, including a 10.6% test failure rate (partly due to difficulties maintaining a continuous power supply), heterogeneous result reporting, and difficulties with supply chain management and sputum transport.^42^

**FIGURE 1:**
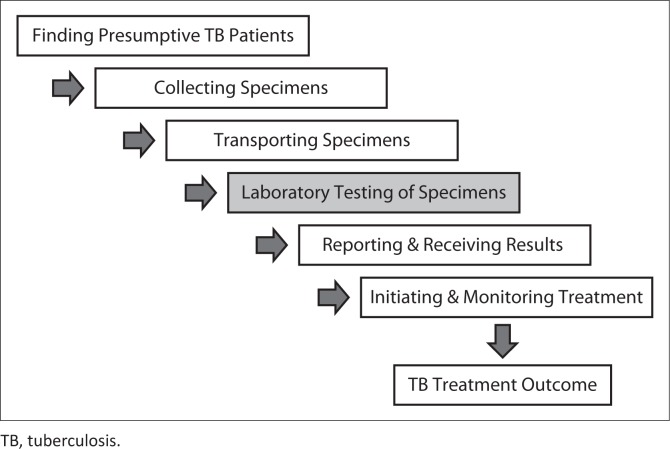
Key steps in the cascade from tuberculosis diagnosis to successful treatment.

**FIGURE 2:**
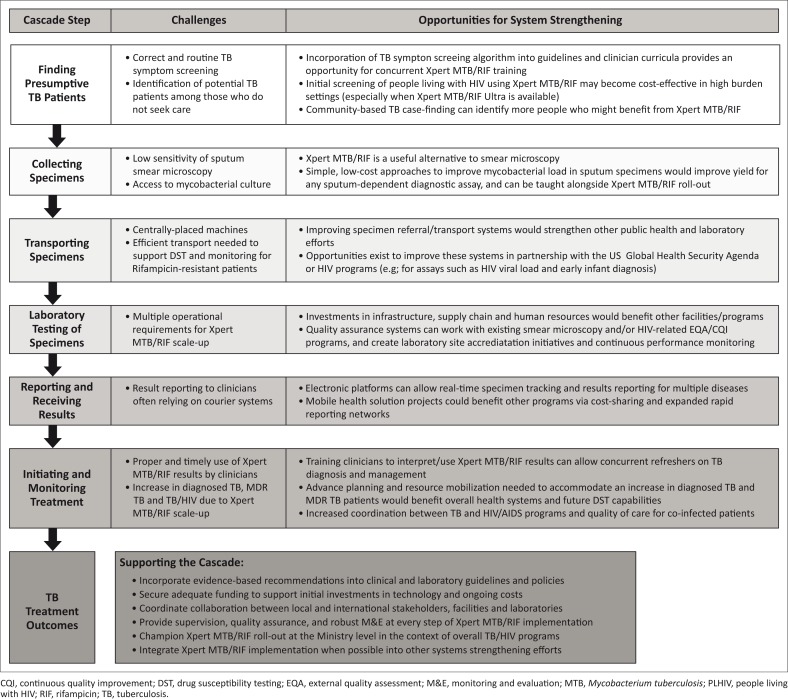
Summary of challenges and opportunities along the tuberculosis care cascade.

These experiences highlight that use of Xpert MTB/RIF technology itself is merely one component within a cascade of activities that must be successful to ensure that all tuberculosis patients are diagnosed and achieve successful and timely treatment and cure (Figure 1). Each process is critical in the programmatic management of tuberculosis (and HIV), and weaknesses in any may minimise the realised utility of any new diagnostic. In addition, they must all be supported by adequate funding, coordination and stakeholders engagement, as exemplified by the varied success of Xpert MTB/RIF implementation thus far. While this technology and newer assays certainly present opportunities for rapid, accurate diagnosis closer to the POC, to maximise their impact in programmatic settings it is crucial that we concurrently optimise other system- and programme-related factors necessary for tuberculosis diagnosis and treatment. Scale-up of Xpert MTB/RIF for diagnosis of tuberculosis in people living with HIV presents a unique opportunity to leverage current tuberculosis and HIV laboratory, diagnostic and programmatic investments, and to coordinate with multiple stakeholders to strengthen laboratory systems, laboratory-programme interfaces, and tuberculosis-HIV programme interfaces overall. Investments in evidence-based interventions at each step along the cascade and within the system as a whole will augment not only the utility of Xpert MTB/RIF, but also the successful implementation of future diagnostic tests (Figure 2).

## Opportunities for system strengthening along the tuberculosis diagnosis and treatment cascade

### Finding presumptive tuberculosis patients

The WHO recommends Xpert MTB/RIF as the initial diagnostic test in individuals with suspected HIV-associated or MDR tuberculosis, which eliminates the additional clinic visit needed to perform sputum microscopy followed by Xpert MTB/RIF if negative.^11,43^ WHO guidelines also recommend that people living with HIV be evaluated at every clinical encounter for cough, fever, weight loss or night sweats, with positive symptom screens prompting further diagnostic evaluation; this screening algorithm has a 79% overall sensitivity (90% in clinical settings) and 50% specificity.^44,45^ Subsequent Xpert MTB/RIF diagnostic testing further increases case detection sensitivity and specificity; however, to test the maximum number of people living with HIV for presumptive tuberculosis, correctly-performed symptom screening is often required first. Universal uptake of the recommended screening algorithm will require incorporation into national guidelines and clinician training in most settings, and provides an opportunity for concurrent sensitisation to Xpert MTB/RIF. Importantly, however, reliance on symptom screening before performing a tuberculosis diagnostic test can miss asymptomatic patients. The potential role for initial tuberculosis screening of people living with HIV using Xpert MTB/RIF (regardless of symptoms) may become cost-effective in high tuberculosis-burden settings, especially with the anticipated roll-out of Xpert MTB/RIF Ultra, which is much more sensitive for smear-negative tuberculosis (94% sensitivity reported preliminarily among smear-negative, culture-positive patients).^46^ This highly-sensitive technology may also empower clinicians to reduce widespread empiric tuberculosis treatment among people living with HIV in the future.

Tuberculosis case detection among people living with HIV can be maximised by strategically placing GeneXpert^®^ machines in facilities and areas with the highest tuberculosis and HIV prevalence. However, even under the best programmatic circumstances, clinical screening algorithms alone may be insufficient to identify tuberculosis among those who do not seek care, are contacts of known tuberculosis patients or are from remote or marginalised populations. Randomised interventions, such as tuberculosis contact tracing, mobile vans, household tuberculosis and HIV counseling, as well as screening reduced tuberculosis prevalence in communities in Zambia, South Africa and Zimbabwe,^47,48^ and active community-based tuberculosis case-finding endeavours, have improved tuberculosis case detection in other low-income settings.^49,50,51,52,53,54^ Such community-based efforts have great potential to identify additional persons at risk for tuberculosis, who can subsequently benefit from diagnostic technologies such as Xpert MTB/RIF.

### Collecting specimens

The sensitivity of smear microscopy for one sputum specimen was 29% in a study of people living with HIV with presumptive tuberculosis in Thailand and Vietnam, and the incremental yield was 7% for two sputum specimens and 2% for three.^55^ Most tuberculosis programmes routinely collect and examine at least two specimens per patient; however, the collective sensitivity of even three sputum smears remains low, and access to mycobacterial culture is limited in most low-resource settings. Xpert MTB/RIF offers a useful alternative, with particularly high sensitivity in sputum smear-positive people living with HIV (95% – 99%).^4^ In one study of patients with smear-negative tuberculosis, the sensitivity of Xpert MTB/RIF for one sample was only 72.5%, but this increased incrementally to 85.1% with two specimens and to 90.2% with three.^56^ In contrast to smear microscopy, however, cost considerations often limit Xpert MTB/RIF to one specimen per individual. Under such resource constraints (and until the validation and widespread availability of Xpert^®^ MTB/RIF Ultra), the collection of a single sputum specimen must at least be optimised.

Although data regarding the impact of sputum quality on tuberculosis diagnosis is sparse and heterogenous,^57^ in theory any method that increases the quality and bacillary load of a specimen should improve diagnostic yield. Mycobacterial load is the most significant predictor of Xpert MTB/RIF-positivity in pulmonary specimens and, in lieu of invasive specimen collection methods, patient instruction can increase microscopic detection of tuberculosis.^58,59,60^ Additional yield can be achieved by supervised, physiotherapy-assisted collection.^61^ Such simple and low-cost approaches are certainly warranted for all sputum specimen collection; even as more sensitive near-POC diagnostics become available, the quality of sputum samples will remain an important predictor of their diagnostic value.^46^ The healthcare worker training necessitated by Xpert MTB/RIF introduction provides an opportunity to re-evaluate and re-direct specimen collection techniques to the benefit of any sputum-dependent diagnostic assay.

Recent advances in transport media can further improve specimen quality. The PrimeStore Molecular Transport Medium^®^ (Longhorn Vaccines and Diagnostics, San Antonio, Texas, United States) inactivates organisms, preserves nucleic acids for molecular detection, and has been shown to enhance tuberculosis detection by Xpert MTB/RIF significantly in samples with low volume and/or bacterial load. Staff familiarity with this medium may facilitate use of other decontaminating transport reagents that preserve organisms for culture.^62,63^

### Transporting specimens

The ability of Xpert MTB/RIF to detect *M. tuberculosis* within two hours is a breakthrough; however, reduced turn-around time is not necessarily sufficient to adequately affect time to diagnosis.^64,65^ In particular, inefficient specimen collection and transport systems have been associated with increased patient attrition, time to appropriate treatment, and culture contamination rates.^7,66^ Improving specimen referral and transport systems is a critical cross-cutting area to target in public health laboratory and tuberculosis systems strengthening efforts worldwide.^67,68^

Although Xpert MTB/RIF may not be a classic POC test, several programmes are choosing to introduce GeneXpert machines in high-volume clinics where presumptive tuberculosis patients are screened and treated, thereby reducing specimen transport needs. However peripheral, low-volume sites may conversely place machines centrally, which increases demand for efficient specimen transport systems. In addition, the WHO recommends that individuals diagnosed with rifampicin-resistant tuberculosis have a specimen referred for laboratory culture and conventional drug susceptibility testing, and are monitored by sputum smear and culture.^13^ Thus, despite the relative success of moving tuberculosis diagnostic capabilities closer to the patient (and even in the context of GeneXpert Omni, which will likely offer true POC tuberculosis diagnosis in the foreseeable future),^69,70^ maintaining and strengthening specimen referral and transport systems remains critical. Recently, US Global Health Security Agenda investments in specimen referral systems, such as safe, standardised sample packaging and shipping using the existing Ugandan early infant diagnosis specimen transportation network, improved the speed and quality of sample transport to national reference laboratories.^71^ The introduction of the Xpert MTB/RIF diagnostic technology to HIV facilities provides a similar opportunity to evaluate specimen transport and referral systems for tuberculosis diagnosis and treatment monitoring – as well as for HIV viral load and early infant diagnosis that are also offered by Cepheid as part of the multi-disease GeneXpert platform^72^ – and to potentially leverage or integrate these systems to ensure timely, safe delivery of biologic specimens to the point of testing.

### Testing specimens

While much excitement surrounding Xpert MTB/RIF has stemmed from its relative user-friendliness, it does have key and sometimes challenging operational requirements. These requirements include an uninterrupted power supply, stable ambient temperatures, waste disposal mechanisms, and equipment security against theft. Supply chains must be reliable, and must control for backlogs in order processing and customs clearance, and limited cartridge shelf life. Modules require annual calibration, machines need routine maintenance, and technical assistance must be readily accessible for trouble-shooting unanticipated challenges.^6,13^ Costs for service and maintenance can be prohibitive, and planning and resource mobilisation must be assured. Early results from Xpert MTB/RIF implementation in nine TB REACH countries indicated a 42% module failure rate (10.6% Xpert MTB/RIF test failure rate), likely due to problems with irregular power, dust build up, overheating and staff quality control.^42,73^ Finally, while each module can process a sample within two hours, backlogs can occur if samples exceed available modules, specimens are batched instead of processed as they are received, or throughput at sites remains below instrument capacity due to staffing or time constraints.^10^ The WHO and others offer recommendations for how to address these issues,^6,11,12,13,42,74^ but implementation of these recommendations requires advanced planning, ongoing coordination, and significant investments in infrastructure.

In addition, Xpert MTB/RIF scale-up requires investments in labour. In one South African primary healthcare setting, Xpert MTB/RIF use increased tuberculosis screening and rapid detection, but POC placement increased logistical responsibilities for the clinic, requiring two to five staff members to provide same-day diagnostic evaluations for 16 patients per day.^40^ In the setting of minimal biosafety concerns, non-laboratory staff are being trained to run the assay in some cases.^23,40,56^ This approach may ease some pressures on limited laboratory human resource capacity; however, emphasis must be placed on testing quality.

Continuous quality improvement for any diagnostic test is critical for ensuring accuracy and reliability, detecting and reducing errors, and ensuring customer satisfaction.^75^ Although the US Centers for Disease Control and Prevention currently provides dried-tube specimen-based proficiency panels to over 400 Xpert MTB/RIF testing sites, dried culture spots have been used by the National Health Laboratory Service in South Africa, and other proficiency testing panels have been developed and assessed, comprehensive external quality assessment programmes for Xpert MTB/RIF remain limited.^76,77,78^ However, tuberculosis laboratories are well-versed in external quality assessment schemes for sputum smear microscopy which, ideally, are run nationally and include blinded re-checking of slides, on-site supervisory visits, panel testing, feedback and corrective action.^79^ These systems are deemed so important that several key WHO laboratory policies are dependent on the presence of a quality-assured smear microscopy network; however, these can be costly and logistically difficult to implement and maintain.^80,81^ Decentralised Xpert MTB/RIF testing shares many parallels with smear microscopy networks and thus, as programmes build quality assurance systems to support its implementation, they should capitalise on the opportunity to work within and improve existing smear microscopy external quality assessment programmes, and to create quality management systems, laboratory and testing site accreditation and certification initiatives. Similarly, as GeneXpert instruments are placed within HIV facilities, plans for ensuring the accuracy and reliability of Xpert MTB/RIF testing should be aligned and/or integrated with systems for HIV-related POC test quality improvement.^75^ Finally, external quality assessment programmes should include supplementation, when possible, with continuous performance monitoring via information systems. This has been accomplished with HIV viral load testing in South Africa, and remote monitoring is a rapidly growing area of interest, supported by GeneXpert and other instrument-based tests.^74,77,82^ HIV programmes offer a well-established model and tools for a stepwise and continuous cycle to plan, implement and sustain quality assurance for POC testing, with emphasis on staff and site certification standards, supervision, and rigorous monitoring and evaluation.^83^ These can be emulated, and strengthened in conjunction with improvements in tuberculosis diagnostic testing continuous quality improvement.

### Reporting and receiving results

The laboratory-clinic interface is often challenged by lack of effective communication. In many settings, courier systems relied upon to transport specimens to the point of testing are also responsible for delivering test results to clinicians. However, the potential impact of rapid diagnostic tests such as Xpert MTB/RIF to improve clinical care cannot be realised if results are not received and interpreted rapidly.^64^ In a 2005 study of smear-positive tuberculosis patients who did not initiate treatment, respondents indicated delays in result receipt as a factor contributing to morbidity and mortality.^7^

The WHO recommends establishment of rapid reporting mechanisms for Xpert MTB/RIF results, including electronic systems, especially in the setting of incompletely decentralised Xpert MTB/RIF availability.^13^ This was highlighted in a notable Cambodian study, where transmitting tuberculosis case-finding results directly to clinicians by text message the day they became available greatly shortened tuberculosis diagnostic delays.^51^ In the previously cited Uganda Global Health Security Agenda project, enhancement of an existing online, open-source communication system to integrate data sources from laboratory, transportation and communication networks allowed real-time tracking of specimens and results.^71^ Prevention of mother-to-child transmission of HIV programmes provide another example of how currently available technologies, such as mobile phones, web-based information systems, and text messaging, can decrease early infant diagnosis result reporting times.^84^ A diverse suite of potential mobile health solutions to expedite Xpert MTB/RIF test results for clinical and programme monitoring are emerging from device manufacturers and third-party innovators, and are increasingly maximising the use of laboratory data transmission via mobile telephony, data storage ‘in the cloud’, interoperability and encryption.^85^ However, unique challenges must be anticipated in terms of data ownership agreements, privacy standards, and the need for technological expertise and infrastructure. Coordination with such existing and planned projects may benefit tuberculosis, HIV and other programmes through cost-sharing and expansion of rapid reporting networks.

### Initiating and monitoring treatment

Assuming presumptive patient identification and specimen collection, transport, testing and result reporting all occur in a rapid and high-quality manner, clinicians receiving tuberculosis diagnostic results are then tasked with making treatment decisions. When Xpert MTB/RIF (or other genotypic) results are discrepant from phenotypic results, clinicians must be trained to interpret them and act based on available information.^12,13^ This training offers the added opportunity to refresh them on tuberculosis diagnosis and management.

After interpreting Xpert MTB/RIF results, clinicians must then see patients through to treatment completion and cure. Due to improved sensitivity of Xpert MTB/RIF over smear microscopy, and its capacity to detect rifampicin resistance, increased drug sensitive and MDR tuberculosis case detection among people living with HIV is anticipated with Xpert MTB/RIF scale-up. In one South African study, although Xpert MTB/RIF introduction reduced the time to MDR tuberculosis treatment initiation, higher case detection paradoxically increased the waiting list for treatment initiation and admission to a tuberculosis specialty hospital.^86^ Appropriate planning and resource mobilisation is thus critical to accommodate this imminent increase in tuberculosis patients, especially for the management of MDR tuberculosis, which requires dedicated facilities or established community-based models of care, specialised staff and stable drug supplies.^6^ Many low- and middle-income countries currently have limited capacity to provide quality MDR tuberculosis management, and scale-up of its treatment without quality control could fuel development of extensively drug-resistant tuberculosis. Since Xpert MTB/RIF does not distinguish between live and dead bacteria, it cannot be used to monitor disease relapse or treatment failure.^14,87^ Conventional tuberculosis microscopy, culture and drug susceptibility testing are still needed to assess for drug resistance and treatment failure; thus, these systems must concurrently be strengthened even as Xpert MTB/RIF use is scaled up.^6,13^ Conversely, future developments in molecular and phenotypic drug susceptibility testing capabilities in response to newly-available pharmacotherapy options may benefit from improvements made to the tuberculosis diagnostic cascade to accommodate Xpert MTB/RIF.^88^

Finally, improved tuberculosis case detection will increase the number of people living with HIV prioritised for antiretroviral therapy – even in the context of new WHO guidelines recommending antiretroviral therapy initiation regardless of immune status – and is also the gateway for other important tuberculosis/HIV interventions including tuberculosis infection control and preventive therapy.^43,89,90^ In many settings, tuberculosis and HIV care and treatment are provided at different locations within parallel systems. Linking co-infected patients to both tuberculosis treatment and antiretroviral therapy therefore requires strengthened coordination and communication between national tuberculosis and HIV programmes, as well as consideration of integrated service delivery.

### Supporting the cascade

Although each element in the cascade between tuberculosis symptom identification and successful treatment must be optimised individually to maximise the impact of Xpert MTB/RIF technology, there are also several overarching requirements. First, it is important that evidence-based recommendations are incorporated into clinical and laboratory guidelines and policies within national tuberculosis and HIV programmes. A recent survey of 22 high tuberculosis burden countries noted that, while 86% had a policy or algorithm to use Xpert MTB/RIF, most implementation was donor-supported, and not considered sustainable.^74^ Adequate funding is clearly crucial to support initial investments in technology, as well as ongoing costs of cartridges, calibration, staff training and supervision. At current concessional prices, a four-module GeneXpert machine with a computer costs $17 000, cartridges $9.98 each, a calibration kit $450, and shipping an average of $1000 ($1 per cartridge). Programmes also need to budget for service, maintenance and extended-warranty costs.^4,12,13^ All things considered, the total costs of investing in Xpert MTB/RIF technology for the first year are an estimated $61 000 per machine, with subsequent annual running costs of around $32 000.^6^ Although shown to be cost-effective in many settings, cost-effectiveness does not necessarily imply affordability, especially in countries with yearly health expenditures often less than $20 per capita.^86^ Despite increases in international donor funding for tuberculosis programmes since 2002, the yearly funding gap was predicted in 2013 to exceed $2 billion by this year.^91^ Given the limited resources but known benefit, Xpert MTB/RIF implementation must be prioritised for maximum ease and impact and, most importantly, integrated whenever possible into other systems strengthening efforts currently underway.^6,20,89^

The second critical factor required to support the tuberculosis diagnostic cascade is increased local and international stakeholder commitment to tuberculosis programmes in general, and coordination of efforts between healthcare sectors, facilities and central governments, and healthcare settings and laboratories. Xpert MTB/RIF has generated significant interest and investment among Ministries of Health, research institutions, and donors. To ensure the success of initial phased implementation projects and national scale-up plans, it is important that tools, innovations and best practices are shared, and that all efforts within countries are coordinated and championed at the ministry level according to national priorities.^92^

Finally, roll-out and national scale-up of projects must be planned in the context of overall tuberculosis and HIV programmes. In particular, as Xpert MTB/RIF use is scaled up, underlying systems must be able to accommodate additional patients expected to be diagnosed using the assay. After stakeholders are coordinated and implementation plans finalised, supplies must be procured, inventories organised, staff trained, and sites prepared for roll-out. Supervision and quality assurance are needed at every step, as is robust monitoring and evaluation – currently not well established – to routinely collect and respond to data on programmatic performance, outcomes and impact, and to assess where instruments are used and where rate- and quality-limiting steps within the cascade are occurring.^92^ Like each individual part of the cascade, these efforts should capitalise on monitoring and evaluation systems already in place, or aim to strengthen those that may benefit from additional investments.

## Conclusion

In 2012, Loveday et al. assessed a typical patient’s journey from diagnosis to treatment in Kwa-Zulu Natal, South Africa, to determine the effectiveness of decentralised care for MDR tuberculosis patients. Although both patient- and health system-related factors resulted in ultimately sub-optimal outcomes, most challenges encountered were due to health systems factors, including poor communication of laboratory results, incorrect provider implementation of clinical guidelines, and inadequate integration of tuberculosis and HIV services. This ‘typical journey’ highlights the fact that weaknesses at any step in the clinical cascade can compound deficits in others.^93^ Conversely, improvements at any step can and should benefit the system as a whole.

Xpert MTB/RIF is a major diagnostic breakthrough, but will only improve patient outcomes if optimally placed and implemented within the context of strong tuberculosis programmes and systems. The roll-out and rapid scale-up of this technology to people living with HIV and others in resource-limited settings offers a unique opportunity to address current challenges to maximise impact on the quality of tuberculosis programmes in general. Ministries of Health, funding agencies and implementing partners should capitalise upon this opportunity by investing in strong, patient-centred health systems, staff and programmes, not only to optimise the success of Xpert MTB/RIF (and other GeneXpert-supported platforms such as HIV viral load testing and early infant diagnosis), but also to allow any future technologies to be seamlessly incorporated and implemented.^73,94^ In particular, Xpert MTB/RIF implementation should be leveraged to strengthen collaborative tuberculosis HIV activities, laboratory networks, and the laboratory-clinic interface. If this is accomplished, the benefits of this diagnostic tool could extend beyond increased tuberculosis case detection and treatment, toward achieving global End TB Strategy goals, improving system-wide capacity for disease detection and control, and promoting global health security.
